# The Mammalian Locus Coeruleus Complex—Consistencies and Variances in Nuclear Organization

**DOI:** 10.3390/brainsci11111486

**Published:** 2021-11-10

**Authors:** Paul R. Manger, Oxana Eschenko

**Affiliations:** 1 School of Anatomical Sciences, Faculty of Health Sciences, University of the Witwatersrand, Johannesburg 2193, South Africa; 2 Department of Physiology of Cognitive Processes, Max Planck Institute for Biological Cybernetics, 72072 Tuebingen, Germany; oxana.eschenko@tuebingen.mpg.de

**Keywords:** noradrenaline, catecholamines, evolution, mammalian brain, subcoeruleus, locus coeruleus, phylogenetic variation

## Abstract

Descriptions of the nuclear parcellation of the locus coeruleus complex have been provided in approximately 80 mammal species spanning the phylogenetic breadth of this class. Within the mammalian rostral hindbrain, noradrenergic neurons (revealed with tyrosine hydroxylase and dopamine-ß-hydroxylase immunohistochemistry) have been observed within the periventricular grey matter (A4 and A6 nuclei) and parvicellular reticular nucleus (A5 and A7 nuclei), with the one exception to date being the tree pangolin, where no A4/A6 neurons are observed. The alphanumeric nomenclature system, developed in laboratory rodent brains, has been adapted to cover the variation observed across species. Cross-species homology is observed regarding the nuclear organization of noradrenergic neurons located in the parvicellular reticular nucleus (A5 and A7). In contrast, significant variations are observed in the organization of the A6 neurons of the locus coeruleus proper. In most mammals, the A6 is comprised of a moderate density of neurons, but in Murid rodents, primates, and megachiropteran bats, the A6 exhibits a very high density of neurons. In primates and megachiropterans, there is an additional moderate density of A6 neurons located rostromedial to the high-density portion. These variations are of importance in understanding the translation of findings in laboratory rodents to humans.

## 1. Introduction

The locus coeruleus was first noted in the human brain by Félix Vicq d’Azyr and described in his 1786 treatise “Traité d’Anatomie et de Physiologie” as a pigmented structure in the rostral hindbrain [1]; however, it wasn’t until 1964 that the locus coeruleus neurons were shown to contain monoamines [2]. It is now known that the locus coeruleus is a complex of neuromodulatory nuclei, present in all vertebrates [3], with neurons that primarily produce the neurotransmitter noradrenaline and are the source of diffuse ascending and descending projections [4,5,6,7,8]. The release of noradrenaline in the terminal fields is correlated with a wide range of functional effects on the targeted cells [9,10,11]. Although laboratory rodent brains have been the most commonly used model to study the structure and function of the locus coeruleus complex, at present, the anatomy of this complex has been examined in around 80 mammal species (Table 1) [2,12,13,14,15,16,17,18,19,20,21,22,23,24,25,26,27,28,29,30,31,32,33,34,35,36,37,38,39,40,41,42,43,44,45,46,47,48,49,50,51,52,53,54,55,56,57,58,59,60,61]. Until recently, the use of tyrosine hydroxylase immunohistochemistry (the rate-limiting enzyme in the synthesis of catecholamines) has been the gold standard for identification of noradrenaline-containing neurons, and the use of this antibody has permitted substantive cross-species analyses. Given the broad range of mammals in which this nuclear complex has been examined and the variations that have been noted, it is of importance to contextualize this comparative research with the more often pursued translational research. It is important to elucidate where, in what lineages, and what changes took place in the nuclear organization of this complex, and how these variations may impact the potential for findings in the intensely studied laboratory rodents [62] to aid our understanding of function and dysfunction of the locus coeruleus complex in humans.

## 2. Location, Nuclear Parcellation, and Nomenclature of the Locus Coeruleus Complex in Laboratory Rodents

**Figure 1:**
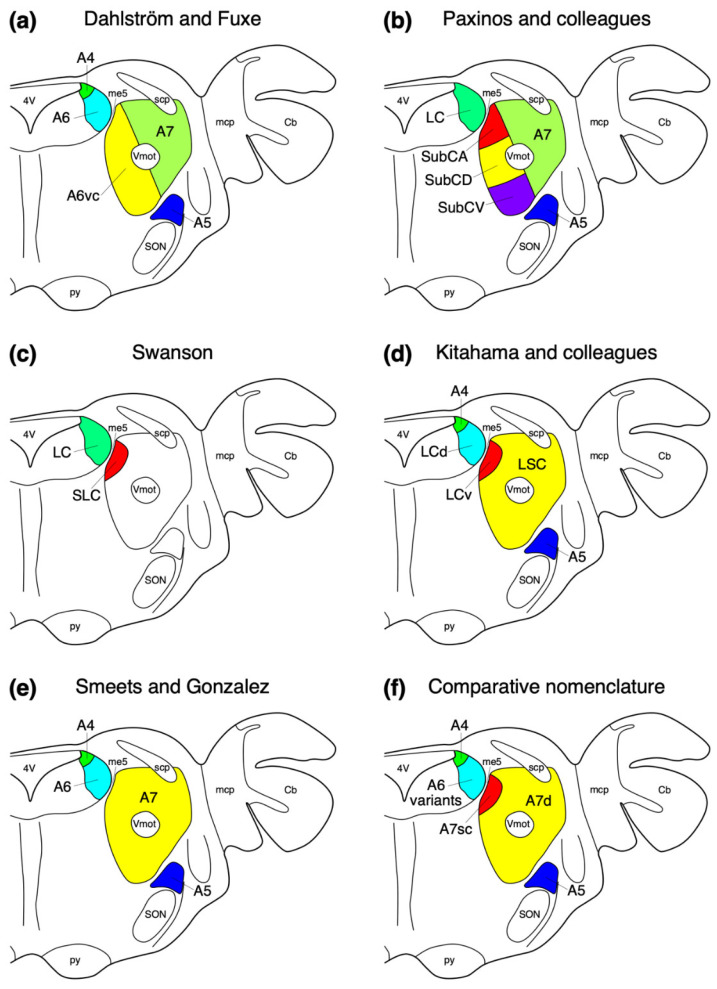
Diagrammatic representations of the parcellation and nomenclature used to describe the locus coeruleus complex in mammalian species in a generalized coronal section through the rostral hindbrain of a mammal. (**a**) The alphanumeric nomenclature applied by Dahlström and Fuxe [2] for the rat brain. (**b**) The combined anatomical and alphanumeric nomenclature applied by Paxinos and colleagues in their rat atlases [63,64]. (**c**) The anatomical nomenclature applied by Swanson [65] in his rat atlases. (**d**,**e**) The mixed anatomical and alphanumeric nomenclature applied to a limited comparative sample by Kitahama and colleagues [61] and Smeets and González [3]. (**f**) The flexible alphanumeric nomenclature adopted in the current study based on observations made in approximately 80 mammal species from across the phylogenetic breadth of mammals (Table 1). Note how across mammal species in general the same broad organization and distribution of noradrenergic neurons is observed, but the nomenclature based on laboratory rodents is not encompassing and required modification to be applicable across mammalian species. 4V—fourth ventricle; Vmot—trigeminal motor nucleus; A4—dorsomedial division of the locus coeruleus; A5—fifth arcuate nucleus; A6—locus coeruleus; A6vc—ventral continuation of locus coeruleus; A7—subcoeruleus; A7d—nucleus subcoeruleus, diffuse portion; A7sc—nucleus subcoeruleus, compact portion; Cb—cerebellum; LC—locus coeruleus; LCd—dorsal locus coeruleus; LCv—ventral locus coeruleus; LSC—locus subcoeruleus; mcp—middle cerebellar peduncle; me5—fifth mesencephalic tract; py—pyramidal tract; scp—superior cerebellar peduncle; SLC—subceruleus nucleus; SON—superior olivary nuclear complex; SubCA—subcoeruleus nucleus, alpha part (SubCα); SubCD—subcoeruleus nucleus, dorsal part; SubCV—subcoeruleus nucleus, ventral part. See text for details of abbreviations used for the portions of the locus coeruleus complex.

In the adult rat (*Rattus norvegicus*) [2] and mouse (*Mus musculus*) [7] rostral hindbrain, the neurons forming the locus coeruleus complex are typically found in both the lateral aspect of the periventricular grey matter (or central grey matter or griseum pontis) and throughout the parvicellular reticular nucleus (Figure 1a). The initial alphanumeric nomenclature applied to the locus coeruleus complex, based on the study of the laboratory rat [2], described two nuclear subdivisions in the periventricular grey matter (A6 and A4 nuclei) and three within the adjacent parvicellular reticular nucleus (A6 ventral continuation, A7. and A5; Figure 1a); however, others have proposed variations of these initial subdivisions and the nomenclature applied (Figure 1) [2,3,63,64,65,66].

## 3. Noradrenergic Neurons (A4 and A6 Nuclei) within the Periventricular Grey Matter of the Laboratory Rodent Rostral Hindbrain

The neurons forming the A4 nucleus of the laboratory rat were described by Dahlström and Fuxe ([2], p. 14) as being located “in the lateral part of the roof of the fourth ventricle, just under the ependyma, ventral to the cerebellar nuclei.” It has been shown that these neurons project to the cerebellum in the rat [67]. They further described ([2], p. 17) the A6 nucleus as “seems to be identical with the locus coeruleus” as defined from architectonic studies, with, “All—or at least practically all—of its closely packed nerve cells belong to the catecholamine-type” ([2], p. 17). The A6 nucleus was observed in the lateral aspect of the periventricular grey matter, extending from the floor of the fourth ventricle to the ventrolateral aspect of the periventricular grey matter. Due to the lack of a distinct anatomical border between the neurons forming the A6 and A4 nuclei of Dahlström and Fuxe [2], others have grouped these as a single nucleus within the periventricular grey matter of the laboratory rodent brain, naming these two nuclei the locus coeruleus (LC) or dorsal locus coeruleus (LCd) (e.g., [7,63,64,65,66]) (Figure 1).

## 4. Noradrenergic Neurons (A5 and A7) within the Parvicellular Reticular Nucleus of the Rostral Hindbrain

Within the reticular region, between one and five noradrenergic neuron clusters have been described (Figure 1). Initially, Dahlström and Fuxe [2] described a column of cells medial to the trigeminal motor nucleus, which was indicated to be a ventral continuation of the A6 nucleus, with cells located lateral to the trigeminal motor nucleus being classified as the A7 group. In addition, they described a cluster of neurons lateral to the superior olivary nuclear complex that was labelled as the A5 nucleus.

Dahlström and Fuxe ([2], p. 17) defined the neurons belonging to the A5 nucleus of the rat as being located “among the fibres of the tractus rubro-spinalis mainly at the level of the caudal and middle third of the nuc. olivaris superior”. The A5 nucleus has been consistently identified in subsequent studies of laboratory rodents, with no specific nomenclature variations (e.g., [7,63,64,65,66]) (Figure 1).

The initial subdivision of the noradrenergic neurons in the parvicellular reticular nucleus identified a column of neurons medial to the trigeminal motor nucleus that were described as, “A row of…cells…observed to pass from the ventral part of the rostral portion of the locus coeruleus in an arch medial to the nuc. motorius n. trigemini down to the cells within group A5” ([2], p. 17/18). In addition, cells observed lateral to the trigeminal motor nucleus were named group A7 (Figure 1a).

The reasons to differentiate these parvicellular reticular noradrenergic neurons from those in the periventricular grey matter (A6 neurons) are the clear anatomical differences, with neurons within grey matter vs. reticular matter, and the presence of the fifth mesencephalic tract between neuronal groups of the grey and reticular matter. This differentiation is supported by studies of connectivity showing differential projection patterns between the noradrenergic neurons located in the periventricular grey matter and those located in the parvicellular reticular nucleus [5,67,68,69,70,71,72] and development [7]. In addition, there is distributional continuity of the parvicellular reticular noradrenergic neurons between the ventral continuation of the A6 and the very laterally placed neurons that in some schemes are considered separately as the A7 group [2,63,64] (Figure 1a,b) despite the lack of clear developmental genetic evidence supporting this division [7].

The parcellation schemes of the noradrenergic neurons have been modified since the initial descriptions, with, for example, Aston-Jones [71], combining the A4, A6, and dorsal-most part of the A6 ventral continuation of Dahlström and Fuxe [2] as the locus coeruleus, while separating the A5 and A7 (being the remainder of the A6 ventral continuation and the A7 group of Dahlström and Fuxe [2]). Within the rat brain atlases of Paxinos and colleagues [63,64], and studies of the mouse brain [7], the A7 cells lateral to the trigeminal motor nucleus are consistent with the initial description, but the A6 ventral column has been termed the subcoeruleus and subdivided into three parts, the subcoeruleus nucleus, alpha part (SubCA/SubCα); subcoeruleus nucleus, dorsal part (SubCD); and subcoeruleus nucleus, ventral part (SubCV). It should be noted that Robertson and colleagues [7] in their study of the mouse brain only describe the SubCD and SubCV. The rat brain atlas of Swanson [65] identifies only the most dorsal portion of the A6 ventral column, labelling this the subceruleus nucleus (SLC), while not specifically identifying the remaining noradrenergic neurons in the parvicellular reticular nucleus (Figure 1). Kitahama and colleagues [66] identify the ventral part of locus coeruleus (A6v), which appears to correspond to the SubCα of Paxinos and colleagues [63,64] and the SLC of Swanson [65], with the remaining cells being ascribed to the locus subcoeruleus (LSC), which appears to correspond to the SubCD, SubCV, and A7 of Paxinos and colleagues [63,64] and Robertson and colleagues [7].

## 5. Location, Nuclear Parcellation, and Nomenclature of the Locus Coeruleus Complex in Other Mammals

**Figure 2:**
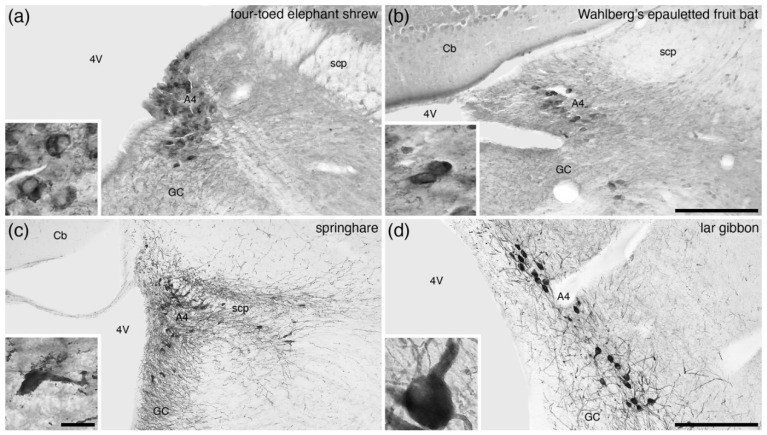
Low-magnification photomicrographs of the dorsolateral division of the locus coeruleus, or **A4** nucleus, revealed with immunostaining for tyrosine hydroxylase, in (**a**) the four-toed sengi (*Petrodromus tetradactylus*) [16], (**b**) Wahlberg’s epauletted fruit bat (*Epomophorus wahlbergi*) [24], (**c**) the springhare (*Pedetes capensis*) [44], and (**d**) the lar gibbon (*Hylobates lar*) [58]. Note that across mammalian species this nucleus has a varied occurrence (Table 1), but when present is consistently located in the same location, that being dorsal to the locus coeruleus proper (A6) within the periventricular grey matter adjacent to the dorsomedial-most part of the superior cerebellar peduncle and cerebellar white matter. Insets in each image show a high-magnification image of the neurons that form the A4 nucleus in each species. In all images, dorsal is to the top and medial to the left. Scale bar in (**b**) = 250 µm and applies to (**a**,**b**). Scale bar in (**d**) = 500 µm and applies to (**c**,**d**). Scale bar in inset (**c**) = 25 µm and applies to all insets. **4V**—fourth ventricle; **Cb**—cerebellum; **GC**—periventricular grey matter of the rostral hindbrain; **scp**—superior cerebellar peduncle.

**Figure 3:**
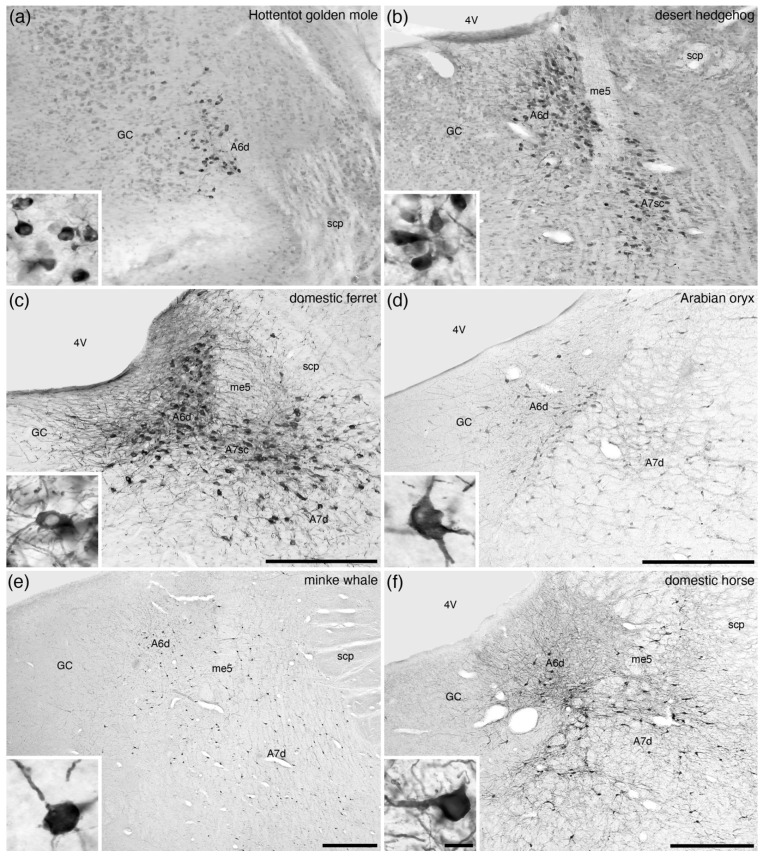
Low-magnification photomicrographs of the locus coeruleus (**A6**), revealed with tyrosine hydroxylase immunostaining, in the brains of one species of Afrotheria, (**a**) the Hottentot golden mole (*Amblysomus hottentotus*) [16], and five species of Laurasiatheria, including (**b**) the desert hedgehog (*Paraechinus aethiopicus*) [19], (**c**) the domestic ferret (*Mustela putorius*) [27], (**d**) the Arabian oryx (*Oryx leucoryx*) [31], (**e**) the minke whale (*Balaenoptera acutorostrata*) [36], and (**f**) the domestic horse (*Equus caballus*) [pers. obs.]. Note how the density of neurons in the locus coeruleus of all these species show the diffuse-type of organization (**A6d**) typical of mammals (Table 1). In all images, dorsal is to the top and medial to the left. Scale bar in (**c**) = 500 µm and applies to (**a**–**c**). Scale bars in (**d**–**f**) = 1 mm and applies to the respective images. Scale bar in inset (**f**) = 25 µm and applies to all insets. **4V**—fourth ventricle; **GC**—periventricular grey matter of the rostral hindbrain; **me5**—fifth mesencephalic tract; **scp**—superior cerebellar peduncle.

**Figure 4:**
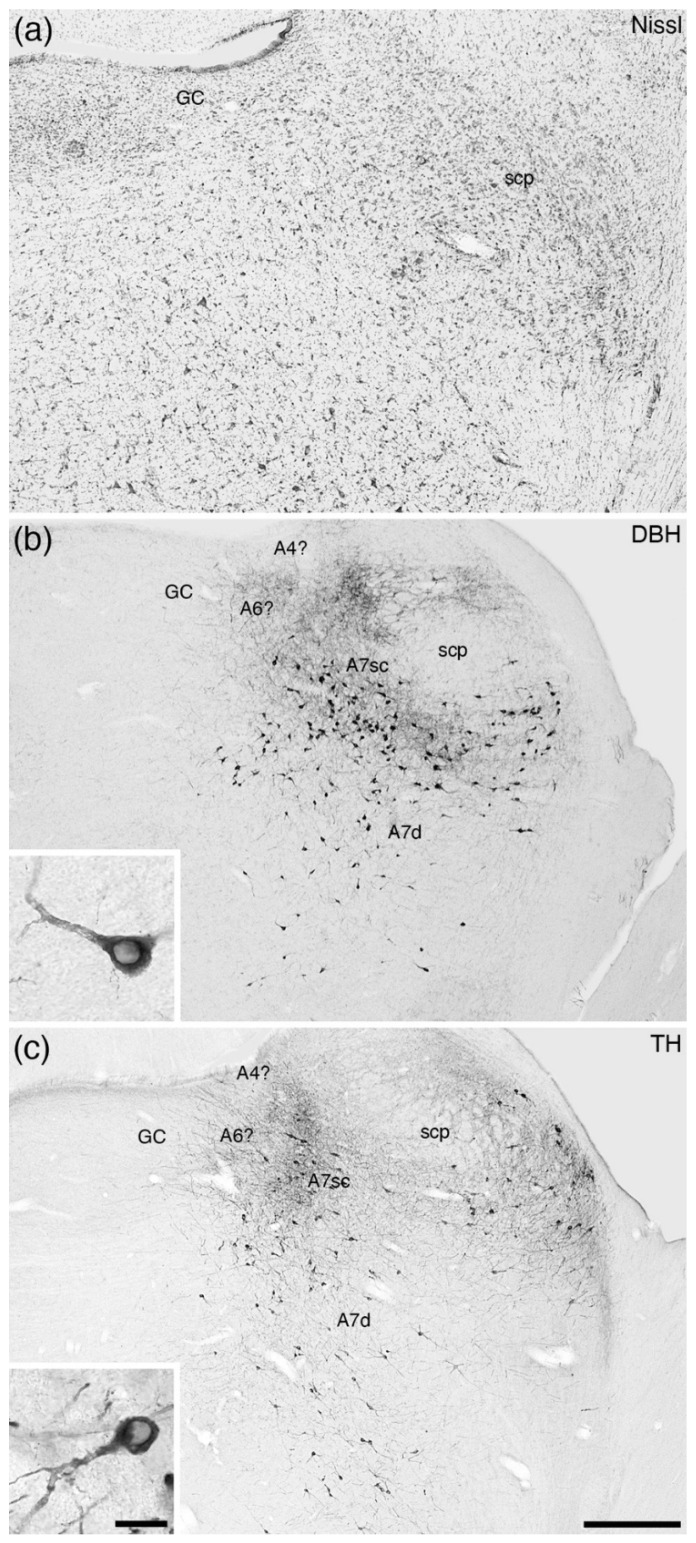
Low-magnification photomicrographs of the subcoeruleus of the tree pangolin [25], revealed with dopamine-β-hydroxylase (**DBH**, (**b**)) and tyrosine hydroxylase (**TH**, (**c**)) immunostaining, to compare with an adjacent Nissl-stained section (**a**). Note the presence of a larger-than-usual compact portion of the subcoeruleus (**A7sc**) and the diffuse portion of the subcoeruleus (**A7d**). No apparent dorsolateral division of the locus coeruleus (**A4?**) or locus coeruleus proper (**A6?**) is observed within the periventricular grey matter of the rostral hindbrain (**GC**). The tree pangolin is the only species in which the absence of a locus coeruleus, A6, has been observed [25]. Insets in (**b**,**c**) show a high-magnification image of the neurons that form the **A7d**. In all images, dorsal is to the top and medial to the left. Scale bar in (**c**) = 500 µm and applies to all images. Scale bar in inset **c** = 25 µm and applies to both insets. **scp**—superior cerebellar peduncle.

**Figure 5:**
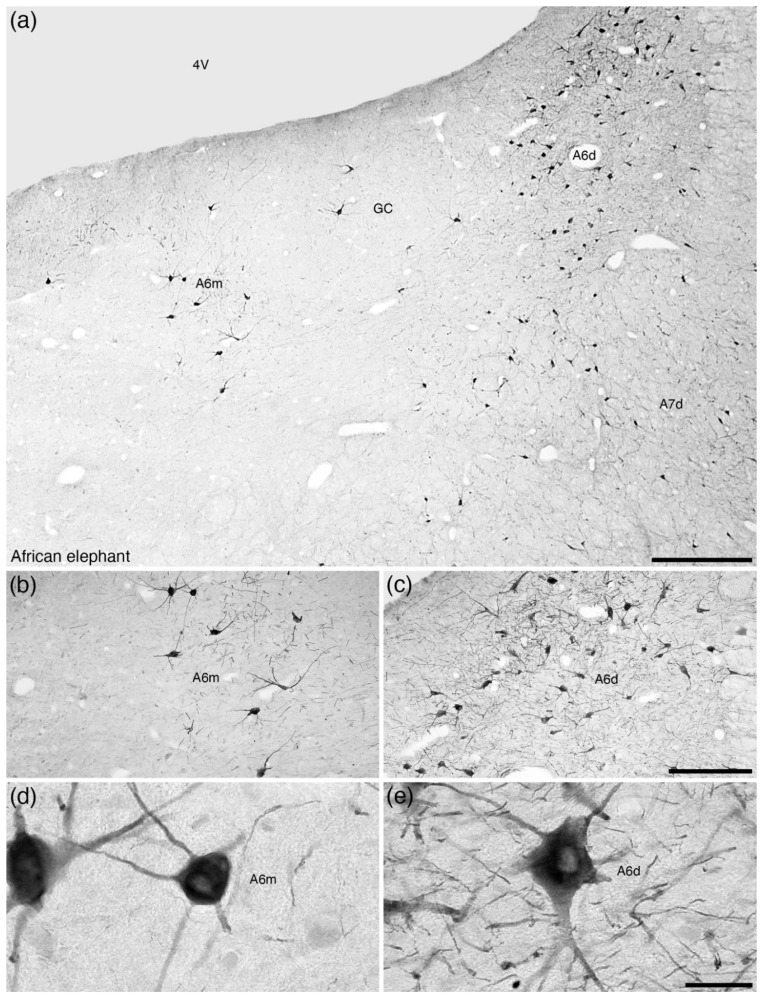
Photomicrographs at various magnifications of the locus coeruleus (**A6**) in the brain of the African elephant (*Loxodonta africana*) [18] revealed with tyrosine hydroxylase immunostaining. The standard mammalian diffuse portion of the locus coeruleus (**A6d**) is present in the ventrolateral periventricular grey matter of the rostral hindbrain (**GC**), but in addition, a medially located cluster of immunopositive neurons, the medial portion of the locus coeruleus (**A6m**), is observed and appears to be a lineage-specific addition to the locus coeruleus complex (**A6**). The neurons of the **A6m** (**b**,**d**) appear to have a slightly more arborized dendritic field than those of the **A6d** (**c**,**e**). In all images, dorsal is to the top and medial to the left. Scale bar in (**a**) = 1 mm. Scale bar in (**c**) = 500 µm and applies to (**b**,**c**). Scale bar in (**e**) = 50 µm and applies to (**d**,**e**). **4V**—fourth ventricle; **A7d**—locus subcoeruleus, diffuse portion; **GC**—periventricular grey matter of the rostral hindbrain.

**Figure 6:**
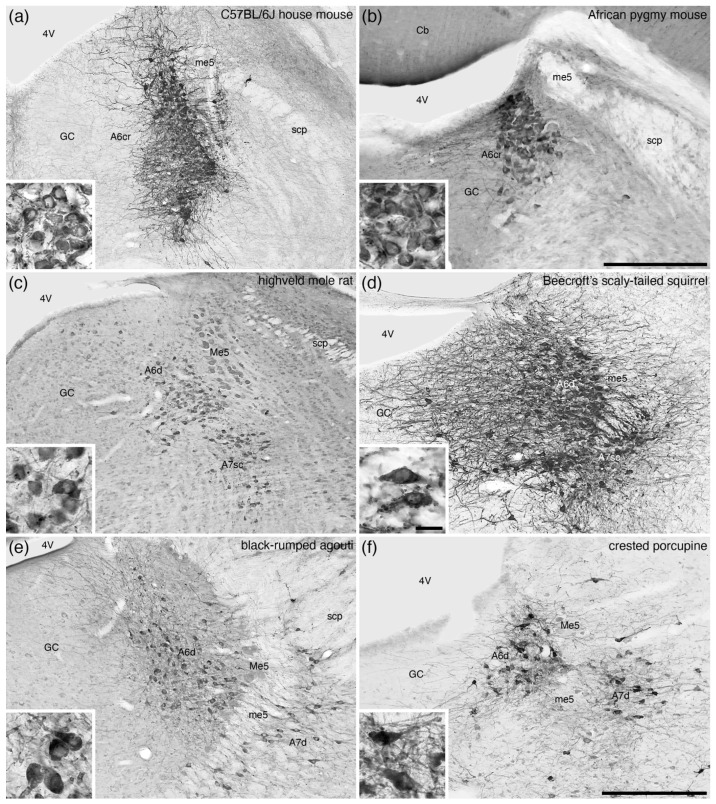
Low-magnification photomicrographs of the locus coeruleus (**A6**), revealed with tyrosine hydroxylase immunostaining, in the brains of six species of rodents, (**a**) the C57BL/6J strain of house mouse (*Mus musculus*) [40,41], (**b**) the African pygmy mouse (*Mus minutoides*) [42], (**c**) the highveld mole-rat (*Cryptomys hottentotus*) [43], (**d**) Beecroft’s scaly-tailed squirrel (*Anomalurus beecrofti*) [44], (**e**) the black-rumped agouti (*Dasyprocta primnolopha*) [pers. obs.], and (**f**) the crested porcupine (*Hystrix africaeaustralis*) [45]. Note that in the two Murid rodents depicted, (**a**,**b**), the neurons forming the **A6** are densely packed and extend dorsally to the floor of the fourth ventricle, forming what we term the compact portion of the locus coeruleus, rodent-type (**A6cr**). In contrast, the non-Murid rodents (**c**–**f**) evince an A6 nucleus that has less densely packed neurons, forming the diffuse portion of the locus coeruleus (**A6d**) as seen in most mammals. Insets show high-magnification images of the neurons from the **A6** in each species. In all images, dorsal is to the top and medial to the left. Scale bar in (**b**) = 250 µm and applies to (**b**) only. Scale bar in (**f**) = 500 µm and applies to (**a**,**c**–**f**). Scale bar in inset (**d**) = 25 µm and applies to all insets. **4V**—fourth ventricle; **A7d**—locus subcoeruleus, diffuse portion; **A7sc**—locus subcoeruleus, compact portion; **GC**—periventricular grey matter of the rostral hindbrain; **Me5**—fifth mesencephalic nucleus; **me5**—fifth mesencephalic tract; **scp**—superior cerebellar peduncle.

**Figure 7:**
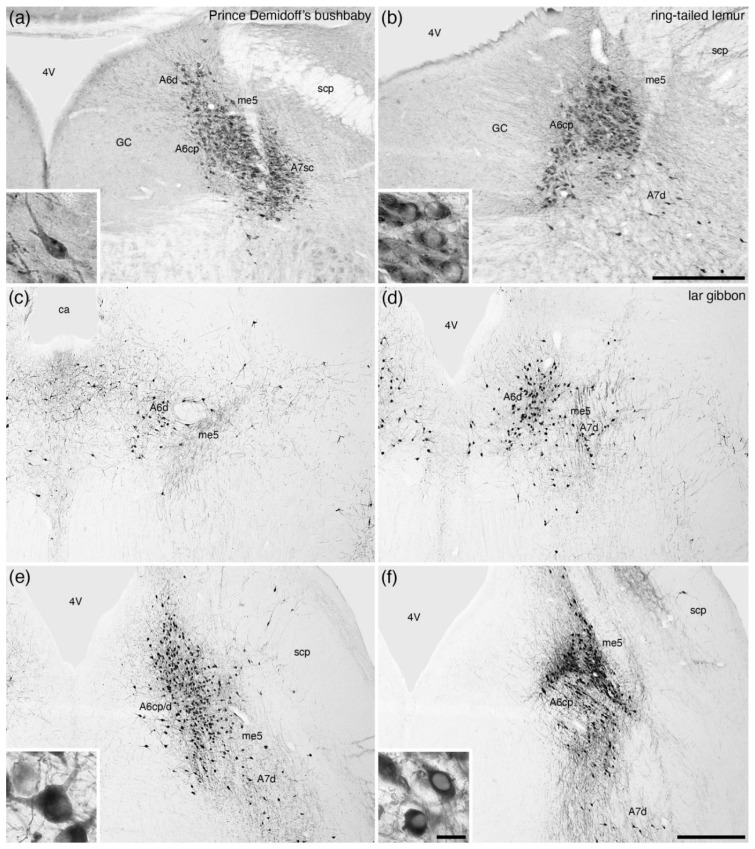
Low-magnification photomicrographs of the locus coeruleus (**A6**), revealed with tyrosine hydroxylase immunostaining, in the brains of three species of primates, (**a**) Prince Demidoff’s bushbaby (*Galago demidoff*) [52], (**b**) ring-tailed lemur (*Lemur catta*) [52], and a rostro-caudal series, with each image being approximately 1 mm apart, through the **A6** of the lar gibbon (*Hylobates lar*) [58]. Note the presence of both diffuse (**A6d**) and compact (**A6cp**, compact portion of the locus coeruleus, primate-type) portions of the **A6** in primates, with the caudal end of the **A6** (**d**) showing the region of highest density of immunostained neurons. Insets in (**a**,**b**,**e**,**f**) show a high-magnification image of the neurons from the **A6d** (**a**,**e**) and **A6cp** (**b**,**d**). In all images, dorsal is to the top and medial to the left. Scale bar in (**b**) = 500 µm and applies to (**a**,**b**). Scale bar in (**f**) = 1 mm and applies to (**c**–**f**). Scale bar in inset (**f**) = 25 µm and applies to all insets. **4V**—fourth ventricle; **ca**—cerebral aqueduct; **GC**—periventricular grey matter of the rostral hindbrain; **me5**—fifth mesencephalic tract; **scp**—superior cerebellar peduncle.

**Figure 8:**
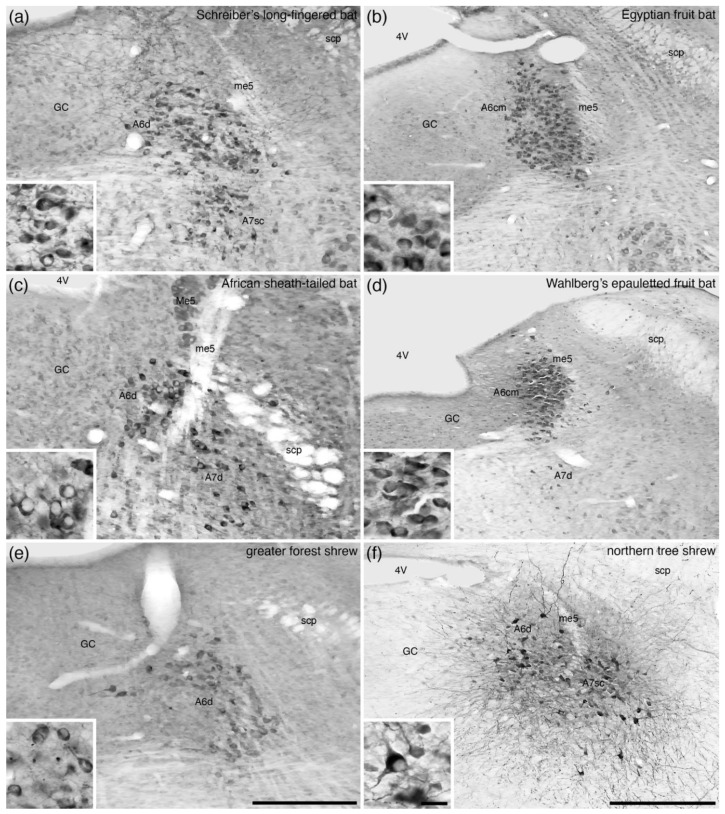
Low-magnification photomicrographs of the locus coeruleus (**A6**), revealed with tyrosine hydroxylase immunostaining, in the brains of two species of microchiropteran bats, (**a**) Schreiber’s long-fingered bat (*Miniopterus schreibersii*) [21] and (**c**) the African sheath-tailed bat (*Coleura afra*) [22], two species of megachiropteran bat, (**b**) the Egyptian rousette (*Rousettus aegyptiacus*) [23] and (**d**) Wahlberg’s epauletted fruit bat (*Epomophorus wahlbergi*) [24], (**e**) the greater forest shrew (*Sylvisorex ollula*) [19], and (**f**) the northern tree shrew (*Tupaia belangeri*) [50]. Note how the density of neurons in the locus coeruleus of the microchiropterans (**a**,**c**), the greater forest shrew (**e**), and the northern tree shrew (**f**) show the diffuse-type of organization (**A6d**) typical of mammals. In contrast, the locus coeruleus in the two species of megachiropterans (**b**,**d**) show a very high density of cells (**A6cm**, compact portion of the locus coeruleus, megachiropteran-type), as well as peripheral regions of low density. This appearance is very similar to what is observed in primates (see Figure 7). In all images, dorsal is to the top and medial to the left. Scale bar in (**e**) = 250 µm and applies to (**a**,**c**,**e**). Scale bar in (**f**) = 500 µm and applies to (**b**,**d**,**f**). Scale bar in inset (**f**) = 25 µm and applies to all insets. **4V**—fourth ventricle; **A7d**—locus subcoeruleus, diffuse portion; **A7sc**—locus subcoeruleus, compact portion; **GC**—periventricular grey matter of the rostral hindbrain; **Me5**—fifth mesencephalic nucleus; **me5**—fifth mesencephalic tract; **scp**—superior cerebellar peduncle.

**Figure 9:**
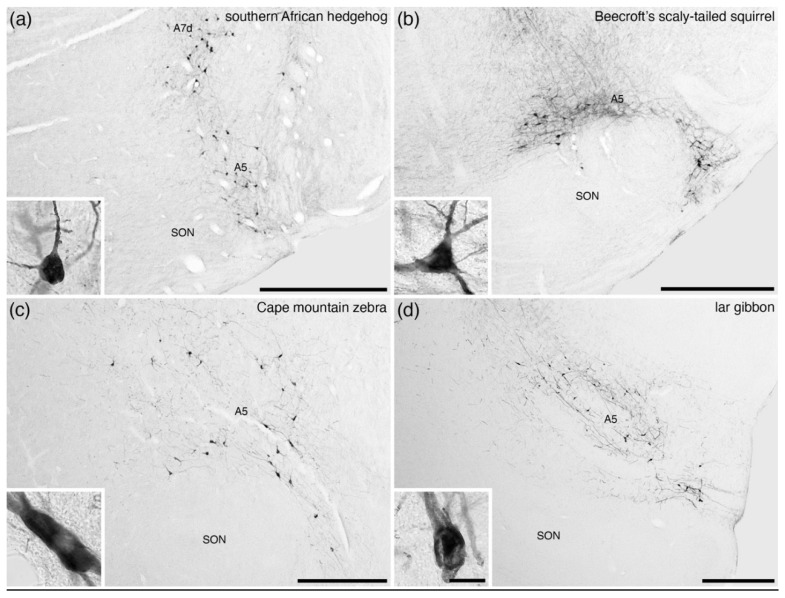
Low-magnification photomicrographs of the fifth arcuate, or **A5**, nucleus, revealed with immunostaining for tyrosine hydroxylase, in (**a**) the southern African hedgehog (*Atelerix frontalis*) [19], (**b**) Beecroft’s scaly-tailed squirrel (*Anomalurus beecrofti*) [44], (**c**) the Cape mountain zebra (*Equus zebra zebra*) [pers. obs.], and (**d**) the lar gibbon (*Hylobates lar*) [58]. Note that across mammalian species this columnar nucleus appears to be invariably present and is consistently located in the same region of the brain, that being ventrolateral to the subcoeruleus, diffuse portion (**A7d**), and dorsolateral to the superior olivary nuclear complex (**SON**). Insets in each image show a high-magnification image of the neurons that form the A5 nucleus in each species. In all images, dorsal is to the top and medial to the left. Scale bars in (**a**–**d**) = 1 mm and apply to each specific image. Scale bar in inset (**d)** = 25 µm and applies to all insets.

**Figure 10:**
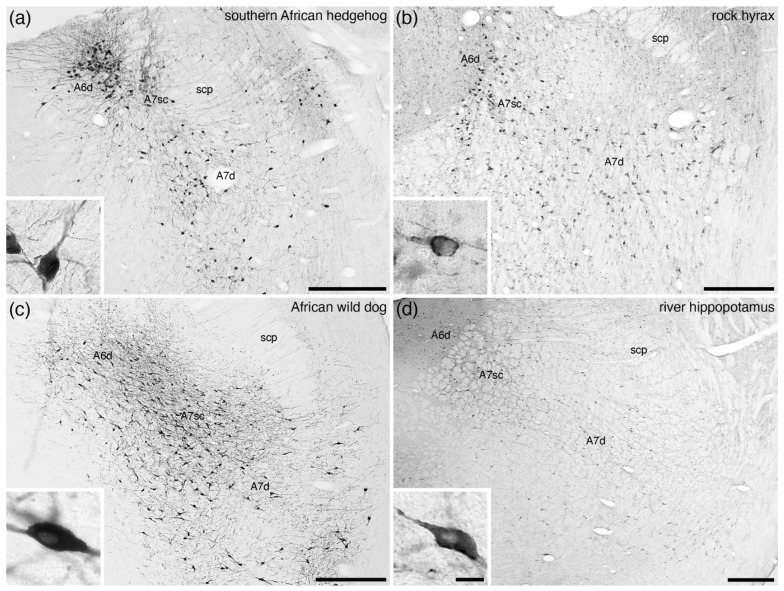
Low-magnification photomicrographs of the subcoeruleus, or A7, region revealed with immunostaining for tyrosine hydroxylase in four mammalian species belonging to the Laurasiatheria superorder of Eutherian mammals, including (**a**) the southern African hedgehog (*Atelerix frontalis*) [19], (**b**) rock hyrax (*Procavia capensis*) [17], (**c**) the African wild dog (*Lycaon pictus*) [pers. obs.], and (**d**) the river hippopotamus (*Hippopotamus amphibius*) [37]. Note the presence of the compact portion of the subcoeruleus (**A7sc**) in the dorsal aspect of the tegmentum, with scattered more widely distributed neurons throughout the parvicellular reticular nucleus forming the diffuse portion of the subcoeruleus (**A7d**). Insets in each image show a high-magnification image of the neurons that form the **A7d** in each species. In all images, dorsal is to the top and medial to the left. Scale bars in (**a**,**b**) = 500 µm and apply to the respective images. Scale bars in (**c**,**d**) = 1 mm and apply to the respective images. Scale bar in inset (**d**) = 25 µm and applies to all insets. **scp**—superior cerebellar peduncle.

**Figure 11:**
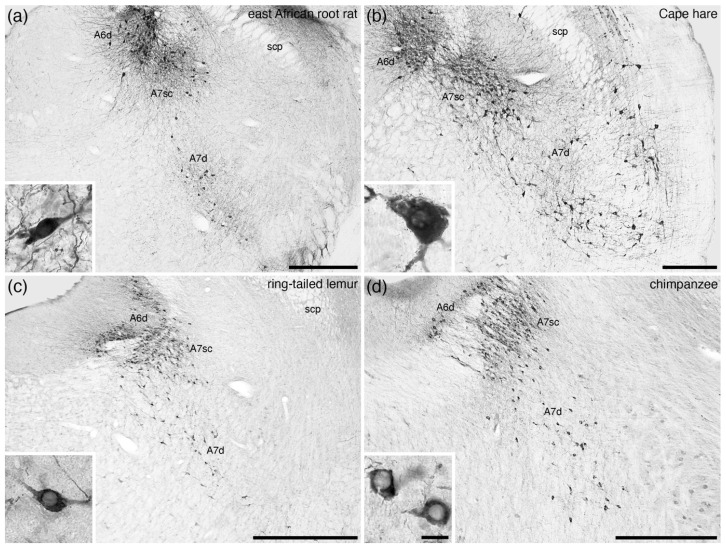
Low-magnification photomicrographs of the subcoeruleus, or A7, region revealed with immunostaining for tyrosine hydroxylase in four mammalian species belonging to the Euarchontoglires superorder of Eutherian mammals, including (**a**) the east African root-rat (*Tachyoryctes splendens*) [pers. obs.], (**b**) Cape hare (*Lepus capensis*) [50], (**c**) the ring-tailed lemur (*Lemur catta*) [52], and (**d**) the chimpanzee (*Pan troglodytes*) [58]. Note the presence of the compact portion of the subcoeruleus (**A7sc**) in the dorsal aspect of the parvicellular reticular nucleus, with scattered more widely distributed neurons throughout the parvicellular reticular nucleus forming the diffuse portion of the subcoeruleus (**A7d**). Insets in each image show a high-magnification image of the neurons that form the **A7d** in each species. In all images, dorsal is to the top and medial to the left. Scale bars in (**a**,**b**) = 500 µm and apply to the respective images. Scale bars in (**c**,**d**) = 1 mm and apply to the respective images. Scale bar in inset (**d**) = 25 µm and applies to all insets. **scp**—superior cerebellar peduncle.

A locus coeruleus complex has been reported in all mammalian species in which the rostral hindbrain has been investigated. Despite this generality, there are variances in the organization of the constituent nuclei that have required the application of a flexible nomenclature to accommodate these within a framework that can be related to the intensely studied laboratory rodents (Figures 1–11). The comparative literature, as detailed below, has identified A4 and A6 nuclei housed within the periventricular grey matter, and A5 and A7 nuclei housed within parvicellular reticular nucleus. Below, we detail the variations that have been observed across species and outline the nomenclature that has been applied to these variations in order to assist in the recognition of potentially homologous and potentially novel nuclei in the various mammalian lineages where the locus coeruleus complex has been studied.

## 6. The A4 Nucleus (Dorsomedial Division of the Locus Coeruleus)

This nucleus, being located in the periventricular grey matter adjacent to the lateral recess of the fourth ventricle and not populated by a large number of neurons, is not always readily observed in the various mammal species that have been investigated (e.g., [32]). The A4 nucleus was not observed in the Prototheria (monotremes) and Metatheria (marsupials) species examined (Table 1). In the Laurasiatheria radiation of Eutherian mammals (Table 1), the presence of an A4 nucleus is varied, being reported in most species of Afrotheria (Figure 2a), the hedgehog lineage of the Eulipotyphla, megachiropteran bats (Figure 2b), the Felidae lineage of carnivores, and the Perissodactyla (Table 1); however, neurons that could be assigned to the A4 nucleus were not observed in the shrew lineage of the Eulipotyphla, the microchiropteran bats, the Philodota, the non-Felidae carnivores, and most Cetartiodactyla (Table 1).

In contrast, the A4 nucleus is consistently observed in all species of the Euarchontoglires radiation of Eutherian mammals (Table 1; Figure 2c,d). While often the A4 is considered part of the A6 (see above), the variability of the presence of A4 neurons across species, primarily conforming to phylogenetic lineages, indicates that it is important to distinguish the A4 as a distinct division of the locus coeruleus complex independent of the locus coeruleus (A6). However, in the species in which the A4 is present, the precise delineation of the boundary between the A4 and A6 neurons is not straightforward, as the A4 neurons appear to be a dorsocaudal continuation of the A6 neurons within the periventricular grey matter. While generally quite a small number of neurons are found in the A4 and their distribution is limited, in both the lar gibbon and chimpanzee, A4 neurons were seen to extend considerably caudal in the ventral white matter of the cerebellum adjacent to the roof of the fourth ventricle [58]. Such an extension of A4 neurons is not observed in non-hominoid primates but does appear to be present in humans [61]. Thus, there is variation in the phylogenetic occurrence of the A4 nucleus and a broader distribution of A4 neurons in hominoids than other mammal species. The reliable delineation of the A4 from the A6 may require connectivity tracing, distinction of specific cell morphologies, or cell-specific molecular labelling [73,74]. The definition of a reliable distinction between A4 and A6 would greatly assist in the interpretation of functional studies in laboratory rodents and primates.

## 7. The A6 Nucleus (Locus Coeruleus)

The locus coeruleus, or A6 nucleus, could be considered the most readily recognizable nucleus of the complex due to its consistent location and substantive density of neurons in mammals; however, the A6 as described in laboratory rodents [2] is atypical in comparison to other mammals [42]. In all mammals studied to date, apart from the tree pangolin [25], noradrenergic neurons are found in the ventrolateral aspect of the periventricular grey matter of the rostral hindbrain, and these are assigned to the A6 nucleus (Table 1). In the majority of mammals studied, the A6 nucleus is reported as being a moderate- to low-density cluster of noradrenergic neurons, which in the comparative neuroanatomical literature has been termed the A6 diffuse (A6d) nucleus (Figure 3; Table 1). Despite this consistent appearance in most mammals, the form of this neuronal cluster varies from being absent in the tree pangolin (Figure 4) [25], to being comprised of relatively few neurons in the rock hyrax [17], having an additional medial nucleus in the African elephant (A6m; Figure 5) [18], being comprised of a single densely packed neuronal cluster (A6c, locus coeruleus, compact portion) in Murid rodents (A6cr, the rodent-type of the A6c, Figure 6a,b) [42], or being comprised of a combination of a high-density cluster bordered by a low-density cluster in primates (A6cp, the primate-type of A6c; Figure 7) and megachiropteran bats (A6cm, the megachiropteran-type of A6c; Figure 8).

To date, the African elephant is the only species examined that shows a distinct topographically separated cluster of noradrenergic neurons in the periventricular grey matter; this cluster, comprised of relatively few neurons, is located medial to the standard A6d nucleus (Figure 5) [18]. Within the order Rodentia, while the majority of species exhibit the typical moderate density of A6 neurons, the A6d (Figure 6c–f; Table 1), the Murid rodents, the lineage to which the commonly used laboratory rodents belong, show a distinctly different organization. In Murid rodents, the neurons forming the A6 nucleus are observed as a densely packed cluster of neurons that spans the dorsoventral extent of the ventrolateral periventricular grey matter (Figure 6a,b). This appearance and organization of the A6 in the Murid rodents appears to be a derived feature of this lineage [42] and indicates that the Murid rodents are unusual when compared to other rodent species, lagomorphs and scandents (Figure 8f).

The appearance of the A6 nucleus in primates is more complex than observed in most other mammals and clearly different from the Murid rodents. In the primate species that have been studied, the rostromedial portion of the A6 region displays a moderate to low density of neurons (akin to the typical mammalian A6d), but the more caudal regions of the A6 in primates shows a densely packed cluster of neurons, the A6cp, that does not span the periventricular grey matter to the floor of the fourth ventricle (Figure 7; Table 1). A very similar organization of the A6 region is observed in the megachiropteran bats, the A6cm (Figure 8b,d; Table 1), but this is not seen in the microchiropteran bats (Figure 8a,c; Table 1). Thus, this primate-like organization of the A6 may have evolved convergently in the primate and megachiropteran lineages, or they may be the result of shared ancestry (see below). The A6 portion of the locus coeruleus complex in mammals displays the most variation in terms of its anatomy, with the species that are used as models for translational research (Murid rodents and primates) showing independently evolved high-density nuclei.

## 8. The A5 Nucleus (Fifth Arcuate Nucleus)

The A5 nucleus has been reported in all mammals studied (Table 1) and is the least variable of all the nuclei of the locus coeruleus complex in terms of location and the low number of neurons across mammalian species (Figure 9). No specific variations have been noted in this nucleus across species and, as such, it is likely that this nucleus is homologous across mammals with its actions likely being analogous.

## 9. The A7 Nuclei (Subcoeruleus)

In the broader comparative context, all noradrenergic neurons located within the parvicellular reticular nucleus of the rostral hindbrain that are not assigned to the A5 nucleus are combined to form the A7 group or subcoeruleus. An additional reason to differentiate the A7 neurons from the A6 neurons is the complete absence of periventricular grey matter noradrenergic neurons in some mammalian species, for example the tree pangolin (Figure 4) [25]. Within this definition of the parvicellular reticular noradrenergic neurons, two distinct populations are consistently observed in the mammals that have been investigated, which include an A7 nucleus subcoeruleus compact (A7sc) and an A7 nucleus subcoeruleus diffuse (A7d) portion (Figure 1f, Figure 10 and Figure 11). The A7sc lies immediately adjacent to the fifth mesencephalic tract in the dorsal-most part of the parvicellular reticular nucleus and is characterized by a moderate to high density of noradrenergic neurons. This portion corresponds to the most dorsal part of the ventral continuation of A6 neurons of Dahlström and Fuxe [2], the subcoeruleus nucleus, alpha part of Paxinos and colleagues [63,64], the subceruleus nucleus (SLC) of Swanson [65], and the ventral part of locus coeruleus (LVc) of Kitahama and colleagues [66]. The A7d noradrenergic neurons are far lower in density and are spread more broadly across the parvicellular reticular nucleus and are topographically continuous with the noradrenergic neurons in the parabrachial region, with these parabrachial neurons being named the A7 group [2,63,64]. The extent of these neurons does vary somewhat across species, but there is no compelling evidence to parcellate the parabrachial noradrenergic neurons from those located more medially [7].

Across all mammalian species studied to date, these two portions of the A7 are consistently reported (Figure 10 and Figure 11; Table 1). The most unusual A7 is found in the tree pangolin (Figure 4) [25], where no noradrenergic neurons are observed within the periventricular grey matter, but the extent of the A7sc and the relative number of neurons comprising the A7sc are expanded in comparison to other mammals. This indicates that these A7sc and A7d nuclei are likely to be homologous nuclei shared by all mammalian species studied to date.

## 10. Consistencies in the Organization of the Mammalian Locus Coeruleus Complex

Given the phylogenetic range of mammalian species in which the locus coeruleus complex has been described (Table 1), it is reasonable to assume that the locus coeruleus complex of mammals is invariably located in the rostral hindbrain. These noradrenergic neurons have been shown to be derived from rhombomeres 1–5 in the developing mouse brain [7], and this is likely to be a common developmental origin for the neurons of the locus coeruleus complex in all mammals. In addition, it is reasonable to state that in mammals, with one known exception [25], the noradrenergic neurons of the locus coeruleus complex are found within the periventricular grey matter and the parvicellular reticular nucleus of the rostral hindbrain. It is also reasonable to posit that the locus coeruleus complex is comprised of several nuclei. Despite this overall similarity, there are distinct structural variances that have required the application of a flexible nomenclature to accommodate these within a framework that can be related to the intensely studied laboratory rodents.

Of the constituent nuclei of the locus coeruleus, the A5 nucleus has been reported in all mammals studied and can be considered a homologous nucleus across species. In rats, the A5 nucleus projects to the intermediolateral cell column of the interramal region of the spinal cord with a specific focus on the sympathetic preganglionic neurons [72]. These projections and the associated functional actions are likely to be a consistent feature in mammals. In addition, the A7sc and A7d nuclei, despite the differing parcellation schemes that have been proposed (Figure 1), appear to be very consistent, probably homologous, features of the locus coeruleus complex across mammals (Table 1). This general consistency in the organization of the noradrenergic neurons within the parvicellular reticular nucleus may be a reflection of their role in the control of the visceral and motor systems [9,10,71]. In contrast to the consistency in the organization of the noradrenergic neurons within the parvicellular reticular nucleus, the organization of those within the periventricular grey appears to be, in an evolutionary sense, more plastic. This in turn may relate to the observations that these neurons, especially those of the A6 nucleus, project to the forebrain and thus they may undergo organizational changes related to evolution of the forebrain [7].

## 11. Variations in the Organization of the Mammalian Locus Coeruleus Complex

The variances noted in the studies undertaken across mammalian species primarily relate to the neurons of the locus coeruleus complex that are found within the periventricular grey matter, specifically the A4 and A6 nuclei as defined by Dahlström and Fuxe [2]. These variances may have important implications for the extrapolation of findings in laboratory rodents to other mammals, particularly humans, where the organization of the A6 is quite different. As several species of the Euarchontoglires superorder have been studied, and the species that occupy phylogenetic positions between the Murid rodents and primates—the non-Murid rodents, lagomorphs and scandents—do not show the compact morphology of the A6, it is clear that the A6cp is a derived feature of the primate (or closely related species, see below) lineage. This increased nuclear complexity in the primate A6 region may also indicate altered projection patterns, functionality, and even internal neurophysiological interactions of the A6 neurons that may be specific to primates. These anatomical (and possibly connectional) variances raise concerns about the direct translatability of the results of functional studies in Murid rodents to the human.

## 12. Gaps in Our Comparative Knowledge of the Mammalian Locus Coeruleus Complex

While the locus coeruleus complex has been described in approximately 80 mammalian species (Table 1), this only represents a small proportion (less than 2%) of mammal species. Despite this, there is considerable understanding of the consistency and variance already obtained, but there are significant gaps in our knowledge that are amenable to further investigation and clarification. Of the approximately 350 Metatherian (marsupial) species, the locus coeruleus complex has only been described in full in two species (Table 1). It is likely that there will be potentially informative variations in the organization of the locus coeruleus complex within the Metatheria. Within the Eutheria, there are specific clades and orders that have not been examined. To date, no descriptions of the locus coeruleus complex have been provided in any Xenarthrans (anteaters, sloths, and armadillos), Tubulidentata (aardvark), Sirenia (sea cows), or Dermoptera (colugos or flying lemurs). The Dermoptera are of particular interest as they are the recognized sister group to the primates (e.g., [75]) and as such occupy an important phylogenetic position in terms of understanding the evolution of the specialized A6 region in primates. Indeed, as the megachiropteran bats exhibit an A6 organization that is very similar to that observed in primates [23,24] and have been proposed to have evolved from the Dermoptera [76], if the Dermoptera were to show an organization of the A6 that is similar to that observed in primates, and the concept that the megachiropterans evolved from the Dermoptera supported, the megachiropterans may become a very useful model species for understanding what may have changed in locus coeruleus function in the primate lineage compared to the Murid rodents. It could also mean that the megachiropterans may be very useful species in translational research regarding the locus coeruleus complex and possibly other regions of the brain.

## 13. How Does the Nuclear Definition of the LC Complex Developed in the Laboratory Rodent Brain Accommodate Variations in Mammalian Species?

It must be openly acknowledged that the laboratory rodents typically studied are but two of several thousand mammal species, and that the commonly used laboratory rodents represent specific strains that have been bred for specific reasons. This may lead to differences in brain structure or function that can undermine the imposition of anatomical nomenclature derived from the rodent brain to other mammals. This is important as there is a growing concern regarding the extrapolation of scientific findings in the laboratory rodents to humans (e.g., [77,78,79]).

When examining species from across the phylogenetic breadth of mammals, the nomenclatures developed in the laboratory rodent brains are to some extent applicable, but in several cases also appear to be untenable. The studies undertaken across mammalian species have mostly employed the alphanumeric nomenclature [2], but the variations noted have required that minor changes and flexibility within this nomenclature were needed in order to be able to describe the variations observed accurately, without inferring potential homologies that may or may not be correct. Indeed, determining the precise homologies of the different portions of the locus coeruleus complex across mammalian species, using an evolutionary developmental, “evo-devo”, approach, is important information that needs to be obtained. Initially, determining the true homologous nuclei of the locus coeruleus in the laboratory rodents and primates is of utmost importance to our understanding of findings made in the laboratory rodents and their relationship to the function and dysfunction of the human locus coeruleus. This determination, and the methods applied, could then be more broadly investigated across mammals, and other vertebrates, in order to improve our understanding of the structure and function of the locus coeruleus complex, leading to an improved understanding of the behaviour and associated neural processes of less commonly studied species. Comparative research has the potential to provide “shortcuts” to develop our understanding of the structure and function of the nervous system (e.g., the giant axon of the squid and the discovery of the action potential is a classic example) that may not be accessible through the more commonly used approach of investigating laboratory rodents and may be the conduit through which we improve the success rate of studies aimed at understanding the function, dysfunction, and treatments of dysfunction of the human brain.

## Figures and Tables

**Table 1:** The nuclear parcellation of the locus coeruleus complex has been described in approximately 80 mammalian species, representing species across the phylogenetic breadth of this class. This table summarizes the results of the studies where the nuclei have been described and the results, as well as indicating where data is unavailable (No data). **A4**—dorsomedial division of locus coeruleus; **A5**—fifth arcuate nucleus; **A6d**—diffuse portion of locus coeruleus; **A6cr**—compact portion of locus coeruleus, rodent-type; **A6cp**—compact portion of locus coeruleus, primate-type; **A6cm**—compact portion of locus coeruleus, megachiropteran-type; **A6m**—medial division of locus coeruleus; **A7sc**—nucleus subcoeruleus, compact portion; **A7d**—nucleus subcoeruleus, diffuse portion; **P**—nucleus present; -—nucleus absent; P.O.—personal observation, ?—data deficient.

Subclass	Clade/ Superorder	Order	Species Number	Scientific Name	Common Name	Nuclei of the Locus Coeruleus Complex									Source(s)
						A4	A5	A6d	A6cr	A6cp	A6cm	A6m	A7sc	A7d	
**Prototheria**		Monotremata	3	*Ornithorhynchus anatinus*	Platypus	-	**P**	**P**	-	-	-	-	**P**	**P**	[12]
				*Tachyglossus aculeatus*	Short-beaked echidna	-	**P**	**P**	-	-	-	-	**P**	**P**	[12]
**Metatheria**	Ameridelphia	Didelphimorphia	108	*Didelphis virginiana*	Virginia opossum	-	?	**P**	-	-	-	-	**P**	**P**	[13]
		Paucituberculata	7	No data											
	Australidelphia	Microbiotheria	1	No data											
		Dasyuromorphia	75	*Sarcophilus harrisii*	Tasmanian devil	-	**P**	**P**	-	-	-	-	**P**	**P**	[14]
		Notoryctemorphia	2	No data											
		Peramelemorphia	24	No data											
		Diprotodontia	137	No data											
**Eutheria**	Xenarthra	Cingulata	22	No data											
		Pilosa	9	No data											
	Afrotheria	Tublidentata	1	No data											
		Macroscelidea	20	*Elephantulus myurus*	Eastern rock elephant shrew	**P**	**P**	**P**	-	-	-	-	**P**	**P**	[15]
				*Petrodromus tetradactylus*	Four-toed sengi	**P**	**P**	**P**	-	-	-	-	**P**	**P**	[16]
		Afrosoricida	30	*Potomogale velox*	Giant otter shrew	**P**	**P**	**P**	-	-	-	-	**P**	**P**	[16]
				*Echinops telfairi*	Lesser hedgehog tenrec	-	**P**	**P**	-	-	-	-	**P**	**P**	P.O.
				*Amblysomus hottentotus*	Hottentot golden mole	**P**	**P**	**P**	-	-	-	-	**P**	**P**	[16]
				*Chrysochloris asiatica*	Cape golden mole	**P**	**P**	**P**	-	-	-	-	**P**	**P**	P.O.
		Hyracoidea	7	*Procavia capensis*	Rock hyrax	**P**	**P**	**P**	-	-	-	-	**P**	**P**	[17]
		Proboscidea	3	*Loxodonta africana*	African bush elephant	-	**P**	**P**	-	-	-	**P**	**P**	**P**	[18]
		Sirenia	4	No data											
	Laurasiatheria	Eulipotyphla	399	*Paraechinus aethiopicus*	Desert hedgehog	**P**	**P**	**P**	-	-	-	-	**P**	**P**	[19]
				*Atelerix frontalis*	Southern African hedgehog	**P**	**P**	**P**	-	-	-	-	**P**	**P**	[19]
				*Erinaceus europeaus*	European hedgehog	?	**P**	**P**	-	-	-	-	**P**	**P**	[20]
				*Crocidura olivieri*	African giant shrew	-	**P**	**P**	-	-	-	-	**P**	**P**	[19]
				*Crocidura cyanea*	Reddish-grey musk shrew	-	**P**	**P**	-	-	-	-	**P**	**P**	[19]
				*Sylvisorex ollula*	Greater forest shrew	-	**P**	**P**	-	-	-	-	**P**	**P**	[19]
		Microchiroptera	1200+	*Miniopterus schreibersii*	Schreiber’s long-fingered bat	-	**P**	**P**	-	-	-	-	**P**	**P**	[21]
				*Chaerophon pumilis*	Little free-tailed bat	-	**P**	**P**	-	-	-	-	**P**	**P**	[22]
				*Hipposideros commersoni*	Commerson’s leaf-nosed bat	-	**P**	**P**	-	-	-	-	**P**	**P**	[22]
				*Cardioderma cor*	Heart-nosed bat	-	**P**	**P**	-	-	-	-	**P**	**P**	[22]
				*Coleura afra*	African sheath-tailed bat	-	**P**	**P**	-	-	-	-	**P**	**P**	[22]
				*Triaenops persicus*	Persian trident bat	-	**P**	**P**	-	-	-	-	**P**	**P**	[22]
		Megachiroptera	190+	*Rousettus aegyptiacus*	Egyptian rousette	**P**	**P**	**P**	-	-	**P**	-	**P**	**P**	[23]
				*Eidolon helvum*	Straw-coloured fruit bat	**P**	**P**	**P**	-	-	**P**	-	**P**	**P**	[24]
				*Epomophorus wahlbergi*	Wahlberg’s epauletted fruit bat	**P**	**P**	**P**	-	-	**P**	-	**P**	**P**	[24]
		Philodota	7	*Manis tricuspis*	Tree pangolin	-	**P**	-	-	-	-	-	**P**	**P**	[25]
		Carnivora	270	*Canis familiaris*	Domestic dog	-	**P**	**P**	-	-	-	-	**P**	**P**	[26]
				*Lycaon pictus*	African wild dog	-	**P**	**P**	-	-	-	-	**P**	**P**	P.O.
				*Mustela putorius*	Domestic ferret	-	**P**	**P**	-	-	-	-	**P**	**P**	[27]
				*Felis cattus*	Domestic cat	**P**	**P**	**P**	-	-	-	-	**P**	**P**	[28]
				*Acinonyx jubatus*	Cheetah	**P**	**P**	**P**	-	-	-	-	**P**	**P**	P.O.
				*Mungos mungo*	Banded mongoose	-	**P**	**P**	-	-	-	-	**P**	**P**	[27]
		Perissodactyla	16	*Equus africanus asinus*	Domestic donkey	**P**	**P**	**P**	-	-	-	-	**P**	**P**	P.O.
				*Equus caballus*	Domestic horse	**P**	**P**	**P**	-	-	-	-	**P**	**P**	P.O.
				*Equus zebra zebra*	Cape mountain zebra	**P**	**P**	**P**	-	-	-	-	**P**	**P**	P.O.
				*Equus quagga*	Plains zebra	**P**	**P**	**P**	-	-	-	-	**P**	**P**	P.O.
				*Tapirus indicus*	Malayan tapir	**P**	**P**	**P**	-	-	-	-	**P**	**P**	P.O.
		Cetartiodactyla	220	*Giraffa camelopardalis*	Giraffe	-	**P**	**P**	-	-	-	-	**P**	**P**	[29]
				*Connochaetes taurinus*	Blue wildbeest	-	**P**	**P**	-	-	-	-	**P**	**P**	[30]
				*Oryx leucoryx*	Arabian oryx	-	**P**	**P**	-	-	-	-	**P**	**P**	[31]
				*Ovis aries*	Domestic sheep	-	**P**	**P**	-	-	-	-	**P**	**P**	[32]
				*Tursiops truncatus*	Bottlenose dolphin	-	**P**	**P**	-	-	-	-	**P**	**P**	[33]
				*Globicephala macrorhynchus*	Short-finned pilot whale	-	**P**	**P**	-	-	-	-	**P**	**P**	[34]
				*Grampus griseus*	Risso’s dolphin	-	**P**	**P**	-	-	-	-	**P**	**P**	[34]
				*Stenella coeruleoalba*	Striped dolphin	-	**P**	**P**	-	-	-	-	**P**	**P**	[34]
				*Stenella frontalis*	Atlantic spotted dolphin	-	**P**	**P**	-	-	-	-	**P**	**P**	[34]
				*Delphinus delphis*	Common dolphin	-	**P**	**P**	-	-	-	-	**P**	**P**	[34]
				*Phocoena phocoena*	Harbour porpoise	**P**	**P**	**P**	-	-	-	-	**P**	**P**	[35]
				*Balaenoptera acutorostrata*	Minke whale	-	**P**	**P**	-	-	-	-	**P**	**P**	[36]
				*Hippopotamus amphibius*	River hippopotamus	-	**P**	**P**	-	-	-	-	**P**	**P**	[37]
				*Lama pacos*	Alpaca	-	**P**	**P**	-	-	-	-	**P**	**P**	[38]
	Euarchontoglires	Rodentia	2200+	*Rattus norvegicus*	Laboratory rat	**P**	**P**	-	**P**	-	-	-	**P**	**P**	[2,39]
				*Mus musculus*	Laboratory mouse	**P**	**P**	-	**P**	-	-	-	**P**	**P**	[40,41]
				*Mus minutoides*	Pygmy mouse	**P**	**P**	-	**P**	-	-	-	**P**	**P**	[42]
				*Tatera brantsii*	Highveld gerbil	**P**	**P**	**P**	-	-	-	-	**P**	**P**	[43]
				*Tachyoryctes splendens*	East African mole-rat	**P**	**P**	**P**	-	-	-	-	**P**	**P**	P.O.
				*Pedetes capensis*	Springhare	**P**	**P**	**P**	-	-	-	-	**P**	**P**	[44]
				*Anomalurus beecrofti*	Beecroft’s scaly-tailed squirrel	**P**	**P**	**P**	-	-	-	-	**P**	**P**	[44]
				*Hystrix africaeaustralis*	Crested porcupine	**P**	**P**	**P**	-	-	-	-	**P**	**P**	[45]
				*Thryonomys swinderianus*	Greater cane rat	**P**	**P**	**P**	-	-	-	-	**P**	**P**	[46]
				*Cryptomys hottentotus*	Highveld mole-rat	**P**	**P**	**P**	-	-	-	-	**P**	**P**	[47,48]
				*Bathyergus suillus*	Cape dune mole-rat	**P**	**P**	**P**	-	-	-	-	**P**	**P**	[48]
				*Georhychus capensis*	Cape mole-rat	**P**	**P**	**P**	-	-	-	-	**P**	**P**	P.O.
				*Dasyprocta primnolopha*	Black-rumped agouti	**P**	**P**	**P**	-	-	-	-	**P**	**P**	P.O.
		Lagomorpha	87	*Oryctolagus cuniculus*	Domestic rabbit	**P**	**P**	**P**	-	-	-	-	**P**	**P**	[49]
				*Lepus capensis*	Cape hare	**P**	**P**	**P**	-	-	-	-	**P**	**P**	[50]
		Scandentia	19	*Tupaia belangeri*	Northern tree shrew	**P**	**P**	**P**	-	-	-	-	**P**	**P**	[50]
				*Tupaia glis*	Common tree shrew	**P**	**P**	**P**	-	-	-	-	**P**	**P**	[51]
		Dermoptera	2	No data											
		Primates	300+	*Galago demidoff*	Prince Demidoff’s bushbaby	**P**	**P**	**P**	-	**P**	-	-	**P**	**P**	[52]
				*Perodicticus potto*	Potto	**P**	**P**	**P**	-	**P**	-	-	**P**	**P**	[52]
				*Lemur catta*	Ring-tailed lemur	**P**	**P**	**P**	-	**P**	-	-	**P**	**P**	[52]
				*Cebuella pygmaea*	Pygmy marmoset	**P**	**P**	**P**	-	**P**	-	-	**P**	**P**	[53]
				*Saimiri sciureus*	Common squirrel monkey	**P**	**P**	**P**	-	**P**	-	-	**P**	**P**	[54,55]
				*Macaca speciosa*	Stump-tailed macaque	**P**	**P**	**P**	-	**P**	-	-	**P**	**P**	[56]
				*Macaca mulatta*	Rhesus macaque	**P**	**P**	**P**	-	**P**	-	-	**P**	**P**	[57]
				*Hylobates lar*	Lar gibbon	**P**	**P**	**P**	-	**P**	-	-	**P**	**P**	[58]
				*Pan troglodytes*	Chimpanzee	**P**	**P**	**P**	-	**P**	-	-	**P**	**P**	[58]
				*Homo sapiens*	Human	**P**	**P**	**P**	-	**P**	-	-	**P**	**P**	[59,60,61]

## Data Availability

All data obtained from published sources.
